# Enoxaparin-Induced Abdominal Wall Haematoma in Pregnancy: A Case Report

**DOI:** 10.7759/cureus.85548

**Published:** 2025-06-08

**Authors:** Nnamdi C Ndukwu, Ritesh Joshi, Irfana Rasool, Sambita Basak

**Affiliations:** 1 Obstetrics and Gynaecology, University Hospital of Northampton, Northamptonshire, GBR; 2 Obstetrics and Gynaecology, Federal Medical Centre, Umuahia, NGA

**Keywords:** enoxaparin, lmwh, pregnancy, rectus sheath haematoma, thromboprophylaxis

## Abstract

Rectus sheath haematoma (RSH) is an uncommon but potentially serious condition in pregnancy, often associated with anticoagulant use. Diagnosis can be challenging as it can mimic other obstetric or surgical emergencies. With the increasing use of low molecular weight heparins in pregnancy, awareness of this complication is essential.

We present a case of enoxaparin-induced RSH in a 42-year-old grand multiparous woman with a history of four previous caesarean sections, who presented at 36 +3 weeks of gestation with signs and symptoms of an acute abdomen. A significant drop in haemoglobin levels, worsening clinical symptoms, and her obstetrics history prompted us to proceed with a caesarean section, during which an RSH was identified.

The caesarean section was completed successfully, and the RSH was managed conservatively. She made a progressive and satisfactory recovery with no further complications.

By sharing our experience, we highlight the diagnostic and management challenges of this condition to improve clinical awareness among obstetricians.

## Introduction

The use of low-molecular-weight heparins (LMWHs) for the treatment of thromboembolism and thromboprophylaxis during pregnancy is in agreement with the Royal College of Obstetricians and Gynaecologists guidelines [1]. Abdominal wall haematomas are rare but emergent complications associated with the use of LMWHs [2]. It mostly results from a rupture of one of the epigastric arteries, leading to an acute bleeding into the rectus sheath [3]. Risk factors include old age, female gender, concomitant anticoagulant use and intense physical activity, previous surgery [4,5]. Abdominal pain with or without a palpable, tender abdominal mass is the most common presentation. This may be followed by anaemia and sometimes haemorrhagic shock [2]. A high index of suspicion is required as physical examination is limited in making a diagnosis [6]. Ultrasounds or computed tomography (CT) scans are required to make a diagnosis [2]. Clinical outcomes range from self-limited bleeding to fatal haemorrhagic shock [7,8]. There is no consensus on the management approach - whether conservative, surgical, or via interventional radiology - for abdominal wall haematomas associated with LMWH use. There is a paucity of reports on abdominal wall haematoma in pregnancy, and diagnosis is challenging as symptoms mimic many other conditions in pregnancy, such as placenta abruption, uterine rupture, ovarian torsion, or acute appendicitis. We, therefore, report a case of a 42-year-old grand multiparous woman on prophylactic dose of enoxaparin who developed a massive abdominal wall haematoma.

## Case presentation

A 42-year-old para 5 with three previous miscarriages presented at 36 weeks and 3 days’ gestation with severe, sudden-onset abdominal pain, preceded by a cough. There was no history of trauma and no vaginal bleeding.

She had caesarean sections four times previously and one normal vaginal delivery. Her body mass index (BMI) was 36.6 kg/m^2^. She was commenced on low-dose Aspirin at 12 weeks of gestation for pre-eclampsia risk reduction and was diagnosed with gestational diabetes mellitus (GDM) in her index pregnancy. She was diagnosed and managed for COVID-19 at 26 weeks gestation and was commenced on prophylactic Enoxaparin at 28 weeks gestation for a venous thromboembolism (VTE) score of 3 for BMI, age and parity.

The patient presented in the early hours of the morning with severe abdominal pain. Maternal observations and cardiotocography (CTG) were normal. The abdomen was tender and tense on the right side. However, a bedside ultrasound scan was done, suspecting a large retroplacental clot of 5 cm x 6 cm. Blood tests showed haemoglobin of 124 g/L, white cell count of 7.6 × 10⁹/L, platelet count of 205 × 10⁹/L and a normal clotting profile. A repeat bedside ultrasound scan was suggestive of a haemorrhagic or torted ovarian cyst of 87.8 x 61.3 mm. Repeat blood tests 7 hours after presentation showed a drop in haemoglobin to 115 g/L. Four units of blood were cross-matched. Risk factors identified in the index patient included female gender, age, four previous surgeries (including cesarean sections), coughing (which could be considered a form of physical exertion, causing contraction of the abdominal wall muscles), and LMWH therapy.

**Table 1:** Blood results from the presentation. Hb, haemoglobin concentration (g/L); WCC, white cell count (10^9/L); Plt, platelet count (10^9/L); CRP, C-reactive protein (mg/L)

	Hb	WCC	Plt	CRP	Clotting profile	Kidney function	Liver function
Presentation	124	7.6	205	6	Normal	Normal	Normal
7 hours after the presentation	115	8.2	175	-	Normal	Normal	Normal
Day 1 post-op	90	10.5	201	8	-		Normal
Day 3 post-op	77	7.3	219	33	-	Normal	Normal
Day 5 post-op	76	4.7	243	33	Normal	Normal	Normal
Day 6 post-op	79	5.2	263	-	-	Normal	Normal
Day 7 post-op	85	5.3	267	-	-	Normal	Normal
Day 12 post-op	119	4.7	221	-	-	Normal	Normal
3 months post-op	134	6.4	216	-	Normal	Normal	Normal

**Table 2:** Reference range and units of measurement.

Measurement	Units	Reference range (adults)
Haemoglobin concentration	g/L	Male: 130-170; female: 120-150
Platelet count	×10⁹/L	150-400
White cell count (WBC)	×10⁹/L	4.0-11.0
C-reactive protein (CRP)	mg/L	<5 (normal)

**Figure 1:**
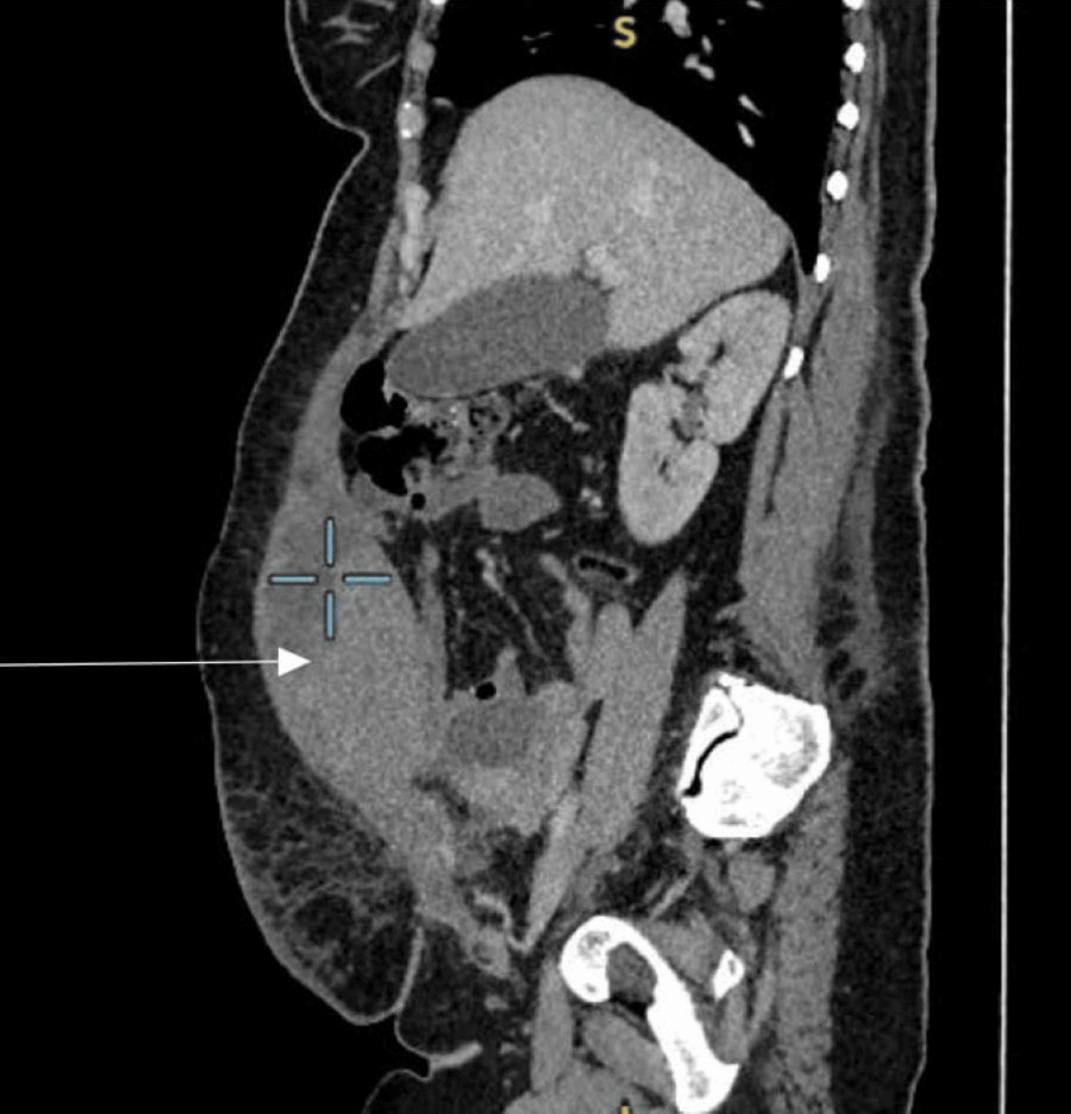
Computed tomography (CT) scan image of the rectus sheath haematoma in sagittal view (arrow indicating the haematoma).

**Figure 2:**
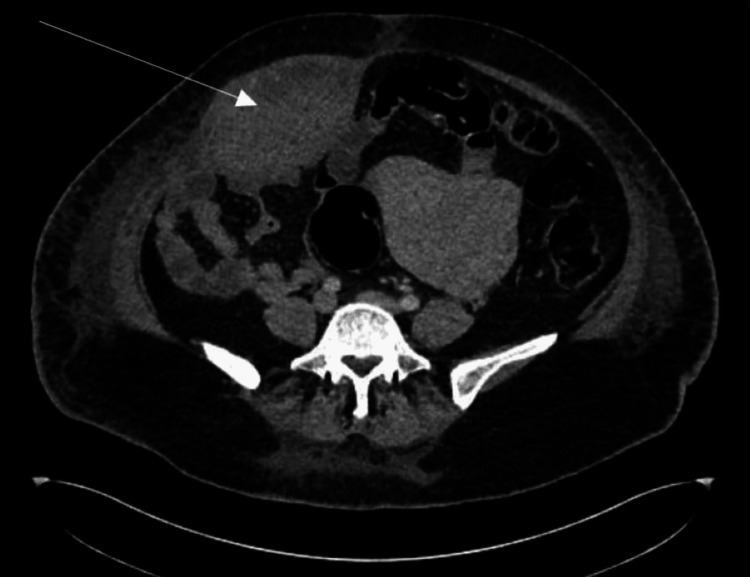
Computed tomography (CT) scan image of the rectus sheath haematoma, coronal view (arrow indicates the haematoma).

Due to worsening pain, the decision was made for an emergency caesarean delivery with the gynaecology team in attendance. She had general anaesthesia due to suspicion of a complex surgery and having had a treatment dose of enoxaparin within the last 12 hours. Intraoperatively, a large haematoma was noted at the right rectus abdominis muscle, extending to the right upper quadrant, measuring approximately 20 × 10 cm. The baby was delivered easily, weighing 3.03 kg (52nd centile). There was no evidence of abruption; normal tubes and ovaries were seen. The surgical team was invited and suggested conservative management. Measured blood loss was 1987 mL. She was admitted to the Intensive Therapy Unit (ITU) post-op due to failed intubation and was later stepped down to the postnatal ward. She was managed conservatively with ongoing clinical observations, analgesics, antibiotics, oral iron, and regular bloods (Tables 1-2). Blood transfusion was not indicated as she was asymptomatic of anaemia with Hb of 90 g/L post-operatively. Enoxaparin was withheld for five days post-op until active bleeding was excluded, based on the risk-benefit discussion within the multi-disciplinary team (MDT). CT abdomen and pelvis was performed on day 5 post-caesarean delivery due to ongoing abdominal pain (Figures 1-2). This showed a large elliptical haematoma within the right rectus sheath (depicted by the arrows in the images) that measured 13 x 7 cm with a speck of high attenuation seen in the inferior aspect of the haematoma and concerning for active bleed from the inferior epigastric artery. Interventional radiologists were involved and suggested to continue with conservative management. Her observations were normal, and the patient felt better: eating, drinking and mobilising well. She received a Ferrinject infusion following a haemoglobin level of 76 g/L, was discharged home on analgesics, and safety-netted on the fifth post-operative day.

**Figure 3:**
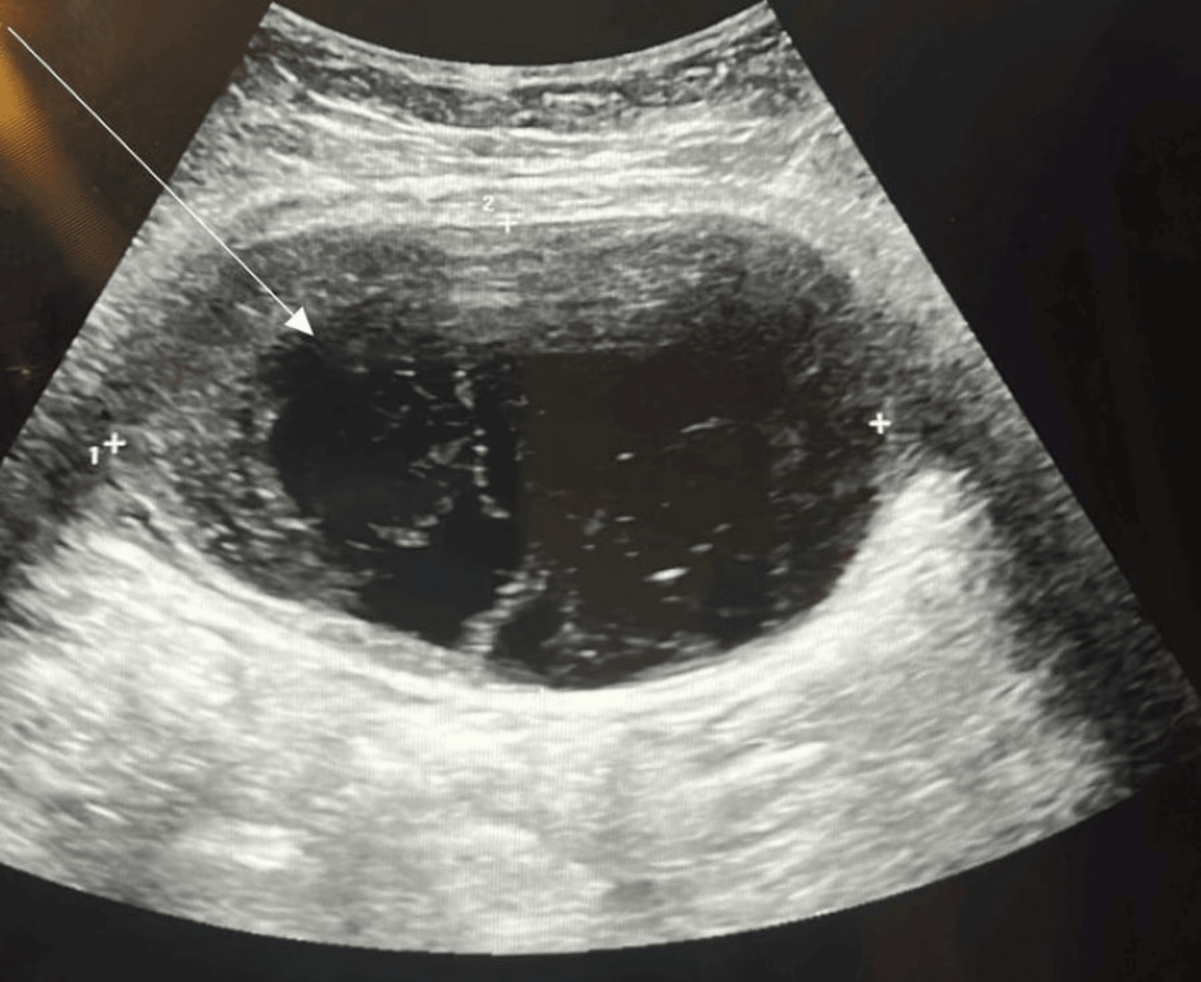
Ultrasound scan image at two weeks post-op (arrow indicates the haematoma).

**Figure 4:**
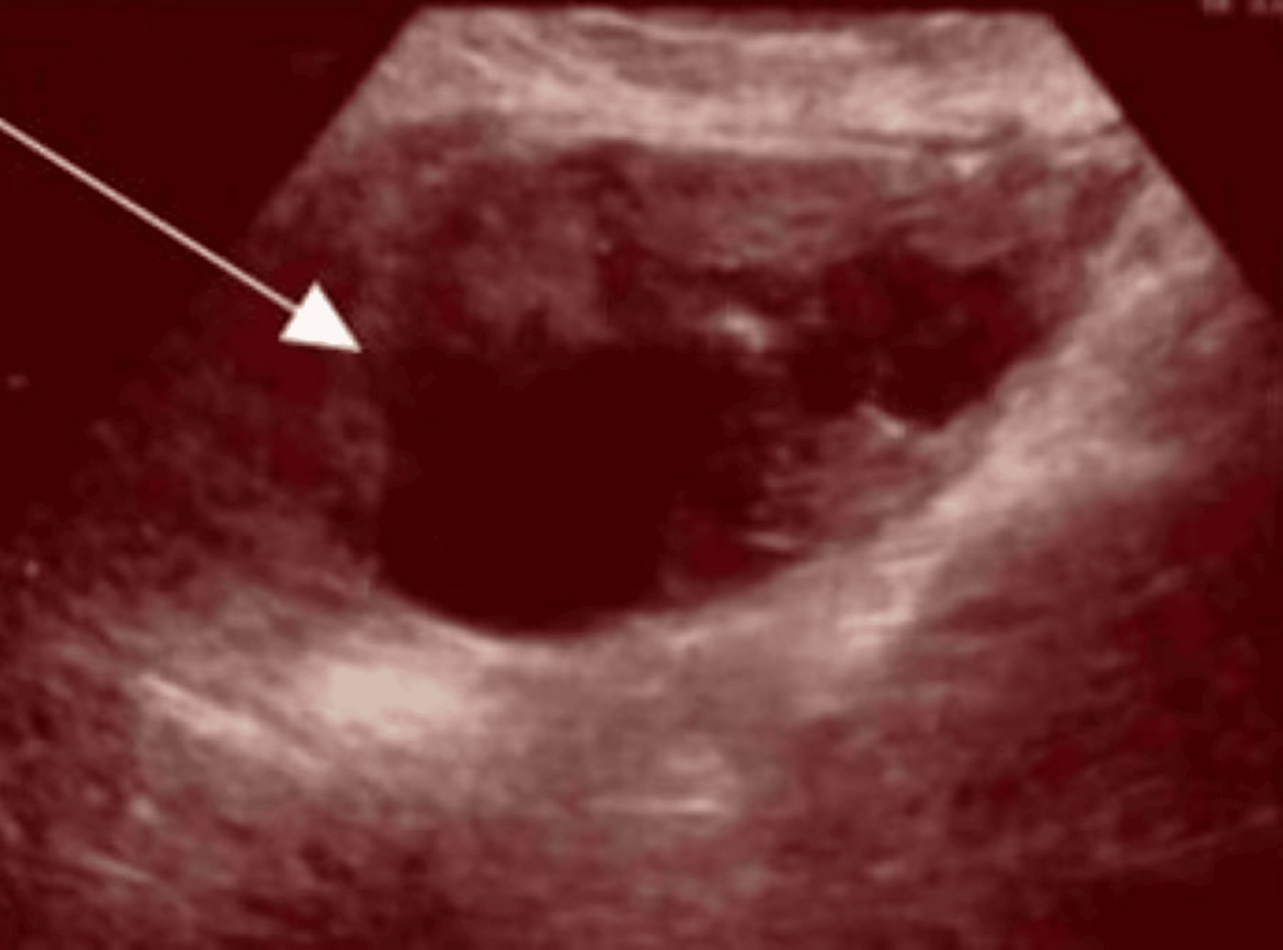
Ultrasound scan image at three weeks and four days post-op (arrow indicates the haematoma).

**Figure 5:**
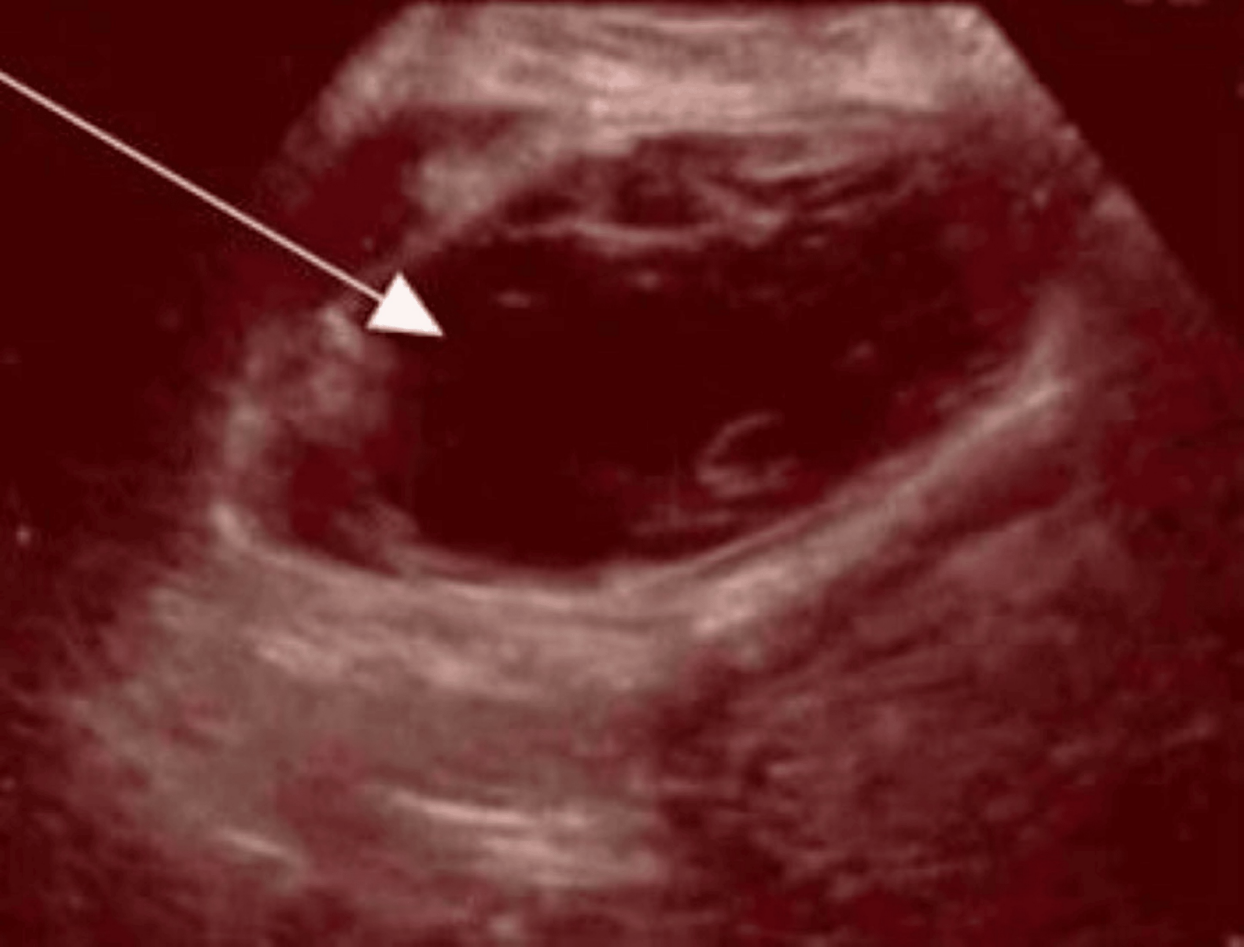
Ultrasound scan image at five weeks post-op (arrow indicates the haematoma).

**Figure 6:**
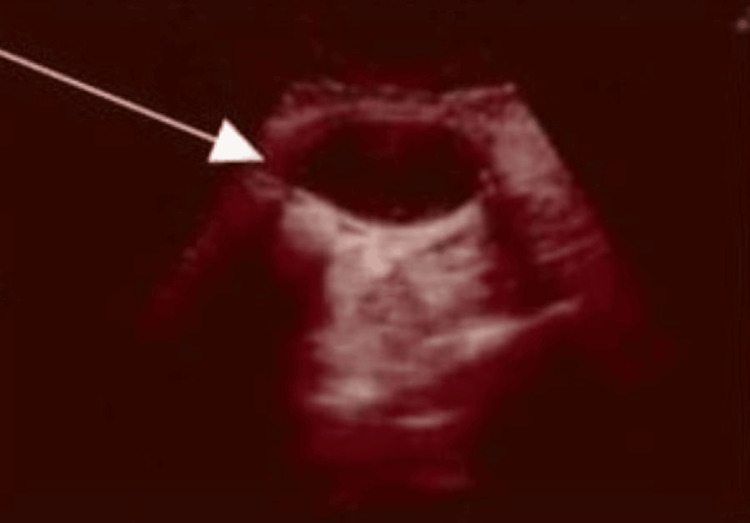
Ultrasound scan image at 11 weeks post-op (arrow indicates the haematoma).

**Figure 7:**
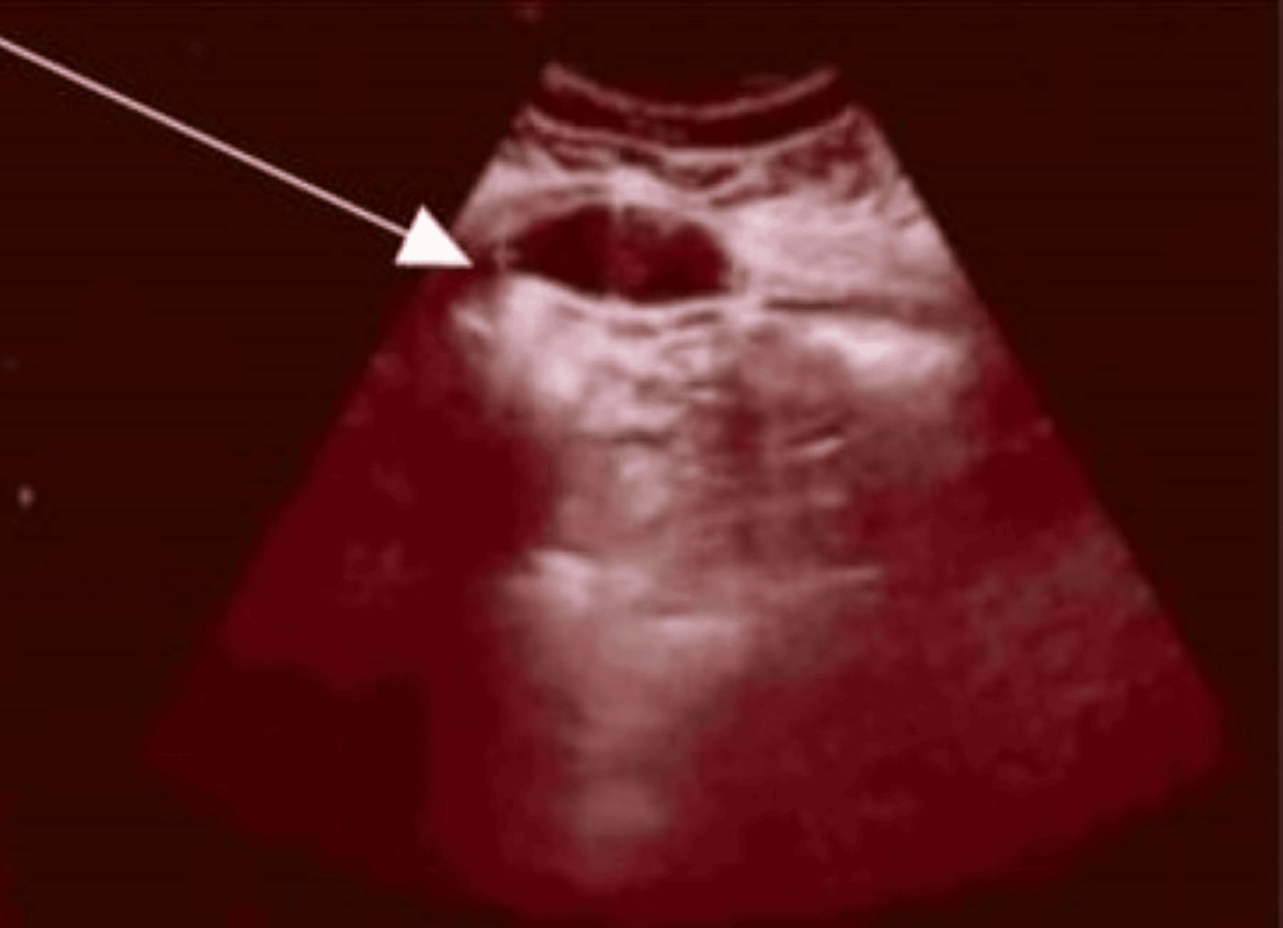
Ultrasound scan image at 23 weeks post-op (arrow indicates the haematoma).

She was followed up in the outpatient clinic at 2, 3, 5, 11 and 23 weeks post-op. An ultrasound scan was performed at each visit, showing a progressive decrease in the size of the haematoma, along with an improvement in symptoms (Table 3; Figures 3-7).

**Table 3:** Progression of haematoma size.

Date	Imaging	Size
5 days post-op	CT abdo-pelvis	130 x 70 mm
2 weeks post-op	Abdominal ultrasound	112 x 96 x 55 mm
3 weeks and 4 days post-op	Abdominal ultrasound	99 x 88 x 86 mm
5 weeks post-op	Abdominal ultrasound	86 x 60 x 55 mm
11 weeks post-op	Abdominal ultrasound	64 x 63 x 32 mm
23 weeks post-op	Abdominal ultrasound	50 x 40 x 20 mm

## Discussion

Acute abdomen in pregnancy constitutes a critical obstetric emergency, requiring immediate evaluation and intervention by an obstetrician. The differential diagnosis for acute abdomen during pregnancy can be categorised into gynaecological and non-gynaecological aetiologies [9]. RSH represents a rare non-gynaecological cause of acute abdomen in pregnant patients. RSH may occur spontaneously or as a result of iatrogenic factors, particularly due to the widespread use of anticoagulant therapy during the antenatal period [10]. In the case presented, enoxaparin was initiated due to antenatal risk factors of maternal age, parity and high BMI.

Patients with RSH typically present with severe, localised abdominal pain, often in a specific quadrant, accompanied by signs and symptoms of hypovolemia that correlate with the extent of blood loss [11]. This was consistent with our patient, who reported pain in the right lower abdomen with a pain score of 8 out of 10, although the initial assessment showed a Maternity Early Obstetric Warning Score (MEOWS) of 0, likely due to the body’s early compensatory mechanisms in response to acute blood loss.

RSH poses a life-threatening risk to both mother and fetus, primarily due to hypovolemia and hypoperfusion resulting from acute blood loss [10]. Consequently, a comprehensive understanding of the signs and symptoms associated with both gynaecological and non-gynaecological conditions is crucial to effectively manage and prevent complications related to this condition.

RSH must be distinguished from other causes of acute abdomen in pregnancy, such as caesarean scar dehiscence, abruptio placentae, ovarian torsion and preterm labour [12]. However, due to its rarity, a diagnosis based solely on clinical evaluation may be challenging [11]. Therefore, the use of imaging modalities like bedside ultrasound, CT, and magnetic resonance imaging (MRI) is essential for accurate diagnosis [13]. In our case, clinical examination revealed a tense and tender right-sided abdomen, prompting the use of ultrasound to evaluate the underlying pathology before proceeding with a caesarean section, followed by a CT scan.

On ultrasound, RSH typically appears as a hypoechoic mass within the abdominal wall [13]. In our case, the initial bedside ultrasound suggested the possibility of a large retroplacental clot performed by a junior doctor, indicative of abruptio placentae, or a haemorrhagic ovarian cyst. This case also shows the challenges to diagnose the condition with bedside ultrasound in an acute setting. While most cases of RSH can be managed conservatively with expectant management, surgical intervention, such as haematoma evacuation, vessel ligation, or drain placement, may be necessary if the maternal or fetal condition is compromised [12]. In our patient, the worsening of symptoms and an acute drop in haemoglobin levels prompted an emergency caesarean section. Upon intraoperative confirmation of RSH, the condition was managed conservatively with follow-up CT scans and ultrasound evaluations.

## Conclusions

RSH is a critical obstetric emergency requiring prompt evaluation and intervention by a senior obstetrician. A thorough patient history, detailed clinical examination, and the use of appropriate imaging modalities are essential for the effective management of RSH. A conservative approach to management may be appropriate in cases where the haematoma is progressively decreasing in size and the patient remains clinically stable. To mitigate the risk of maternal and fetal complications, including preterm delivery, clinicians should maintain a high index of suspicion for RSH in pregnant patients on LMWH who present with localised abdominal pain and signs of blood loss, enabling timely diagnosis and appropriate intervention.
